# Exploiting the Symmetry of the Resonator Mode to Enhance PELDOR Sensitivity

**DOI:** 10.1007/s00723-014-0621-8

**Published:** 2014-12-06

**Authors:** Enrico Salvadori, Mei Wai Fung, Markus Hoffmann, Harry L. Anderson, Christopher W. M. Kay

**Affiliations:** 1 London Centre for Nanotechnology, University College London, 17-19 Gordon Street, London, WC1H 0AH UK; 2 Institute of Structural and Molecular Biology, University College London, Gower Street, London, WC1E 6BT UK; 3 Chemistry Research Laboratory, Department of Chemistry, University of Oxford, South Parks Road, Oxford, OX1 3TA UK

## Abstract

Pulsed electron paramagnetic resonance (EPR) spectroscopy using microwaves at two frequencies can be employed to measure distances between pairs of paramagnets separated by up to 10 nm. The method, combined with site-directed mutagenesis, has become increasingly popular in structural biology for both its selectivity and capability of providing information not accessible through more standard methods such as nuclear magnetic resonance and X-ray crystallography. Despite these advantages, EPR distance measurements suffer from poor sensitivity. One contributing factor is technical: since 65 MHz typically separates the pump and detection frequencies, they cannot both be located at the center of the pseudo-Lorentzian microwave resonance of a single-mode resonator. To maximize the inversion efficiency, the pump pulse is usually placed at the center of the resonance, while the observer frequency is placed in the wing, with consequent reduction in sensitivity. Here, we consider an alternative configuration: by spacing pump and observer frequencies symmetrically with respect to the microwave resonance and by increasing the quality factor, valuable improvement in the signal-to-noise ratio can be obtained.

## Introduction

Electron paramagnetic resonance (EPR) spectroscopy has made many important contributions in elucidating functional aspects of biological machines [1]. Photosynthesis is one of the most extensively covered topics: in particular, functional aspects of electron transfer reactions and energy dissipation were derived from magnetic resonance measurements [2–4]. Most remarkably detailed structural predictions on the arrangement of pigments in the reaction center of photosynthetic organisms could be made on the basis of such data [5–7]. Metal–organic cofactors in proteins are another class of systems that garner advantage from EPR studies. Examples include: nitrogenases [8]; heme proteins [9]; and hydrogenases [10, 11].

On the other hand, the advent of the spin-labeling technique and the introduction of PELDOR (Pulsed ELectron DOuble Resonance, also known as DEER) spectroscopy, together, have revolutionized the applicability of EPR to structural problems in biology, by extending the EPR methodology to proteins lacking intrinsic paramagnetic centers or intermediates [12–14]. Using two different microwave frequencies, PELDOR can provide long-range structural information by detecting the magnitude of the dipolar interaction between pairs of spin labels. The beauty and the strength of the PELDOR approach is that it is neither hampered by the size of the system considered nor does it require long-range order in the sample. Indeed, EPR is particularly suitable when large and complex macromolecules are investigated [15, 16]. Although more sensitive than nuclear magnetic resonance, EPR still needs a high spin concentration to deliver measurements within a reasonable timeframe (less than 24 h) and high-quality data for reliable analysis. When achieving high spin concentration is challenging, careful optimization of all the experimental parameters is mandatory. Recently, considerable efforts have been dedicated to improve the experimental sensitivity, such as: exploiting light-induced electron spin polarization [17], performing the measurements at higher microwave frequencies [18–20], introducing alternative pulse sequences [21, 22], using a combination of higher microwave frequencies and unusual spin labels [23], employing dual mode resonators [24, 25], or stitching together measurements obtained with complementary pulse sequences [26].

**Fig. 1:**
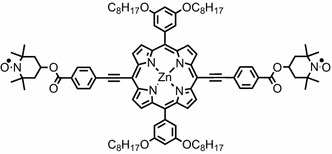
Chemical structure of the bis-TEMPO terminated porphyrin model compound

Apart from the sample, a successful distance measurement based on the dead time-free four-pulse approach [13, 14] performed using a commercially available 9 GHz spectrometer depends on two aspects: optimal inversion by the pump pulse and sensitivity at the detection (observer) frequency. Typically, a measurement is performed with the pump frequency at the center of the microwave resonance: a 12 ns pump pulse allows for optimal inversion and consequently for the deepest modulation depth. Concomitantly, the observer frequency is up-shifted by 65 MHz. This has two consequences for the latter: (i) higher microwave power must be employed to generate the same B_1_ field; (ii) the sensitivity decreases. Here, we show that by placing the two microwave frequencies symmetrically with respect to the center of the microwave resonance (*ν*_center_ ± 32.5 MHz) and optimizing the quality factor (*Q*), the sensitivity of the measurement can be increased without compromising the integrity of the data. To experimentally prove the validity of this alternative approach, we used a stable and rigid bi-radical based on a porphyrin ring terminated with two TEMPO moieties. The chemical structure of such model compound is reported in Fig. 1.

## Experimental

### Resonator Quality Factor: Model and Experimental Validation

The *Q* of a resonator characterizes the ratio between the energy stored and the energy loss per cycle. For convenience, it may be expressed as the ratio between *f*, the central frequency of the resonance, and Δ*f*, the half-power bandwidth: *Q* = *f*/Δ*f*. The theoretical dependence of the resonator resonance against *Q* was modeled assuming a Lorentzian shape for the resonance centered at 9.6 GHz.

The experimental cavity resonance was recorded at variable *Q* using a HP 8722D Network Analyzer. The unloaded resonances were recorded in the range 150 < *Q* < 4,000 at room temperature. The experimental curves were fitted to a Lorentzian function using the custom equation option in the Matlab curve fitting toolbox.

### Sample Preparation

The bi-radical model compound used for this study is based on zinc-porphyrin moiety covalently linked to two TEMPO (2,2,6,6-Tetramethyl-1-piperidinyloxy) radicals, Fig. 1. It was synthesized through Sonogashira coupling of 4-iodobenzoic acid TEMPO ester to alkyne-terminated porphyrin [27]. The molecule was dissolved in dry toluene (50 µM), transferred into a 4-mm EPR tube and sealed under vacuum after several freeze–pump–thaw cycles. The dipolar coupling between the two TEMPO moieties of such bi-radicals has previously been characterized in detail in [27].

### PELDOR Measurements

PELDOR experiments were performed at 50 K on a Bruker ELEXSYS E580 spectrometer operating at 9.6 GHz equipped with an ER 4118 X-MD5 resonator, Oxford Instruments continuous flow cryostat (CF935) and ITC503 temperature controller. The 4-pulse PEDLOR sequence used was *π*/2(*ν*_obs_) − *τ*_1_ − *π*(*ν*_obs_) − *t*′ − *π*(*ν*_pump_) − (*τ*_*l*_ + *τ*_*2*_ − *t*′) − *π*(*ν*_obs_) − *τ*_2_ − echo, where the *π*/2 observer pulse length was 16 ns. The pump pulse length was 12 ns. *τ*_1_ was 200 ns, while the long interpulse delay (*τ*_2_) was 2,000 ns. The PELDOR time traces have a resolution of 8 ns. Each scan includes phase cycling to remove the receiver offset, and nuclear modulation averaging to remove residual ESEEM (Electron Spin Echo Envelope Modulation) contributions. All data are the average of nine scans. For each measurement, the microwave channels’ amplitudes were optimized, whereas the receiver video gain and all other parameters were fixed.

The time traces were analyzed using the program DeerAnalysis 2013 [28]. The background was corrected by a homology three-dimensional fit and the distance distributions were evaluated according to worm-like chain (WLC) model [28] as previously reported for this model system [27].

## Results

**Fig. 2:**
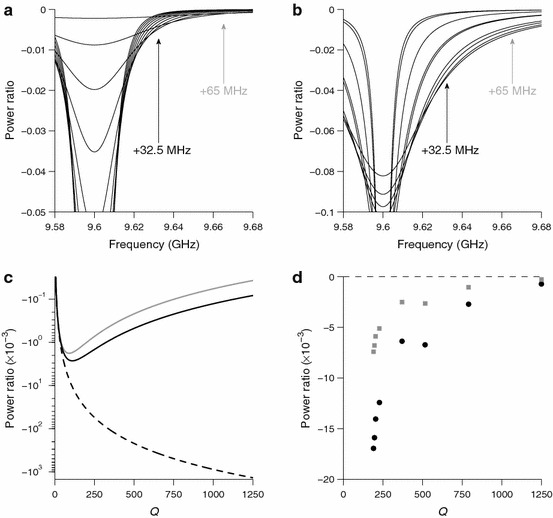
**a** Model dependence of the resonator resonance at varying *Q* (center frequency = 9.6 GHz, 50 < *Q* < 550). The resonance is represented by a series of normalized Lorentzian functions. *Arrows* highlight the *ν*_center_ + 32.5 MHz and *ν*_center_ + 65 MHz frequency offsets. **b** Measured dependence of the resonance at varying *Q* for the unloaded Bruker MD5 resonator at RT. The *curves* presented are the Lorentzian fits onto the recorded traces (190 < *Q* < 1,250). The center of the resonance has been shifted to a reference value (9.6 GHz) for better comparison. *Arrows* highlight the *ν*_center_ + 32.5 MHz and *ν*_center_ + 65 MHz frequency offsets. **c** Dependence of the amplitude of the *model* resonance at different *Q*’s at *ν*_center_ (*dashed*), *ν*_center_ + 32.5 MHz (*black*) and *ν*_center_ + 65 MHz (*gray*) offsets with respect to the center. **d** Dependence of the amplitude of the *measured* resonance at different *Q*’s at *ν*_center_ + 32.5 MHz (*black*) and *ν*_center_ + 65 MHz (*gray*) offsets with respect to the center

Figure 2a shows the dependence of an ideal (normalized Lorentzian) microwave resonance over the range 50 < *Q* < 550. As the *Q* decreases, the resonance changes from being deep and narrow to being shallow and broad until it completely disappears as *Q* approaches zero. Accordingly, the microwave field generated at the center of the resonance drops monotonically, as shown in Fig. 2c (dashed line). Note, however, that away from the center, the microwave field generated is nearly zero at high *Q* and it goes through a maximum as *Q* decreases before going back towards zero as *Q* approaches zero.

Besides the standard positioning of pump and observer frequencies (i.e., pump set at the resonance frequency, observer shifted by +65 MHz), we envisage an alternative configuration for PELDOR, which could enhance sensitivity while maintaining the position of the observer frequency 65 MHz higher than the pump frequency. It entails placing the two frequencies symmetrically within the microwave resonance (i.e., *ν*_center_ ± 32.5 MHz). The price to be paid for this enhancement is that the pump frequency is no longer at the center of the microwave resonance, so more microwave power is required to achieve the same inversion efficiency. Figure 2c also depicts plots of the microwave intensity at *ν*_center_ + 32.5 MHz and *ν*_center_ + 65 MHz versus *Q* (solid lines). The maxima are found at *Q* = 110 and *Q* = 90, respectively. Noteworthy, however, is that the maximum amplitude for an offset of 32.5 MHz is approximately 50 % larger than for an offset of 65 MHz. Hence, not only can a larger microwave field be generated with the smaller offset, but also the EPR sensitivity will be higher.

To validate this idealized Lorentzian model experimentally, the microwave resonance of a Bruker dielectric cavity (ER 4118 X-MD5) was measured for different *Q*. The raw data, collected in reflection mode (dB scale), were converted to power ratio to ease the comparison with the simulated data. The experimental curves were fitted to Lorentzian functions and the relative fits are reported in Fig. 2b. Similar to Fig. 2c, Fig. 2d depicts plots of the microwave intensity at *ν*_center_ + 32.5 MHz and *ν*_center_ + 65 MHz versus *Q*. Note that, as opposed to the model resonance, the Lorentzian fits are not normalized (i.e., the area underneath each curve is not constant): the fits had to be scaled in amplitude to match the experimental data. Unfortunately, it is not possible to provide data for *Q* lower than 190 due to background reflections that prevented reliable fitting. Nevertheless, in agreement with the model, not only do the experimental conversion factors show a trend with a maximum for both offsets at lower *Q*, but also the maximum measured conversion factor is approximately 50 % larger for a frequency of *ν*_center_ + 32.5 MHz compared with *ν*_center_ + 65 MHz, Fig. 2d.

**Fig. 3:**
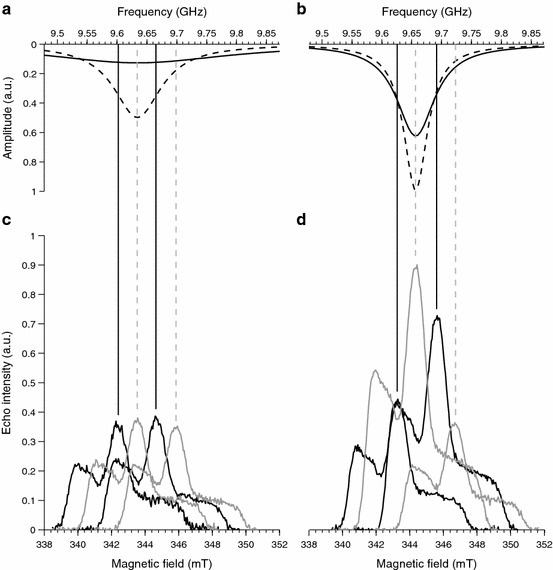
*Upper panels*, reconstruction of the sensitivity profiles (*solid lines*) and simulation of the resonator mode (*dashed lines*) with **a** *Q* = 100 and **b** *Q* = 200 as described in the main text. The *vertical lines* correspond to microwave frequencies used to set up the PELDOR experiments. The *lower panels* report the EDFS spectra recorded at each frequency with **c** *Q* ≈ 100 and **d** *Q* ≈ 200. The amplitude of the microwave channel was varied at each frequency to maximize the signal amplitude. Color code: *gray lines* correspond to the standard configuration (pump = *ν*_center_; observer = *ν*_center_ + 65 MHz); *black lines* correspond to the symmetric configuration (pump = *ν*_center_ − 32.5 MHz; observer = *ν*_center_ + 32.5 MHz)

To test the predicted sensitivity enhancement, we used a model system (Fig. 1) that has been characterized in detail previously [27]. We first measured the echo detected field-swept (EDFS) spectra to map the sensitivity of the resonator at the frequencies relevant to a PELDOR experiment: *ν*_center_; *ν*_center_ ± 32.5 MHz; and *ν*_center_ + 65 MHz. These measurements were repeated for two different *Q*: the lowest *Q* achievable with our Bruker dielectric cavity (nominal *Q* ≈ 100) and a slightly higher *Q* (nominal *Q* ≈ 200). The *Q* is the number given by the built-in *Q* *indicator* in the Xepr program, at 50 K for the loaded resonator. Although these numbers may not be accurate, their usage will allow other investigators to compare results. The shapes of the microwave resonance for *Q* = 100 and *Q* = 200 are illustrated in Fig. 3a and 3b (dashed lines), respectively.

The EDFS spectra recorded with *Q* ≈ 100 and *Q* ≈ 200 at the four microwave frequencies are depicted in Fig. 3c and Fig. 3d, respectively. To compensate for the frequency dependence of the B_1_ field, the microwave power was optimized to maximize the echo intensity at each frequency and for each *Q*. In the case of *Q* ≈ 100, the spectra show similar intensities indicating that the sensitivity is not strongly affected by the position in the microwave resonance, at this low *Q*. Conversely, when considering *Q* ≈ 200, the four spectra show markedly different intensities demonstrating that a relatively small frequency shift has a considerable effect on sensitivity. Thus, when the observer frequency is placed at *ν*_center_ + 32.5 MHz with a *Q* ≈ 200, an EDFS amplitude of approximately twice the other configurations is observed. It is this enhancement that we exploit in the PELDOR measurements below.

We note that the relative intensity of the central peaks of the EDFS spectra may be used to reconstruct the frequency dependence of the sensitivity by fitting them to a Lorentzian line shape. Figure 3a, b (solid lines) shows the results of this procedure. Such fits are characterized by a *Q,* which we refer to as the *Sensitivity*-*Q*. This is related to the actual *Q* of the microwave resonance. In our case, *Sensitivity*-*Q’s* of 30 and 130 were estimated for the *nominal* *Q*’s of 100 and 200, respectively. Figure 3a and b also compares the *Sensitivity*-fit (solid line) with the resonance mode (dashed line). Note that the latter are pure Lorentzian simulations and they are just to illustrate the link between these two parameters. It is reasonable that the *Sensitivity*-*Q* should be lower than the actual *Q* because the microwave power was adjusted to maintain constant B_1_ across the frequency range.

**Fig. 4:**
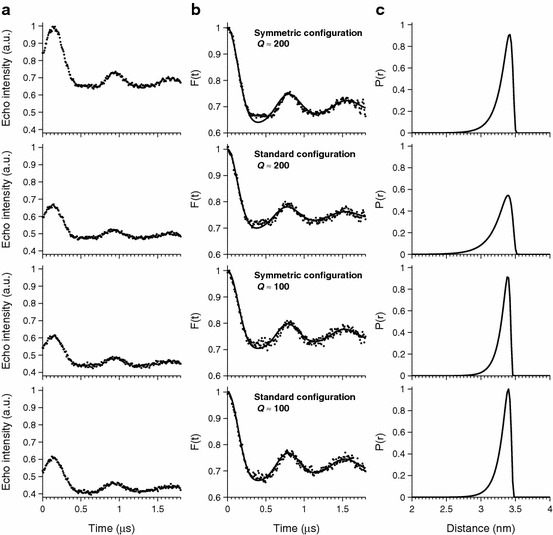
**a** Experimental PELDOR traces: for ease of comparison, the most intense trace has been normalized to one and all the other traces have been scaled accordingly. **b** Background-corrected dipolar evolution data (*dots*) and the fits to the PELDOR data obtained by WLC model implemented in DeerAnalysis (*solid lines*) [27] and **c** corresponding distance distributions

Figure 4 depicts the PELDOR traces for both the standard configuration (pump at *ν*_center_; observer at *ν*_center_ + 65 MHz) and the symmetric configuration (pump and observer at *ν*_center_ ± 32.5 MHz). The model compound investigated has an inter-spin distance of 3.4 nm, corresponding to a dipolar frequency of about 1.5 MHz and has previously been analyzed using the WLC model [27].

Figure 4a shows the echo intensity as a function of the pump pulse delay. The trace recorded with *Q* ≈ 200 and symmetric configuration has almost 40 % larger signal compared to the three other traces. Nevertheless, the noise levels in all four spectra are similar implying a clear gain in signal-to-noise ratio for the symmetrical configuration with *Q* ≈ 200.

Figure 4b shows the background-corrected dipolar evolution and fits thereto using the WLC model. All four PELDOR traces display similar dipolar frequency and similar modulation depth, indicating that the relative position of pump and observer frequencies and *Q* factor do not affect significantly the measurement outcome. After baseline correction, the gain in signal-to-noise ratio for symmetric configuration with *Q* ≈ 200 is clearly visible. In turn, this gain allows the more complex profile of the dipolar evolution curve to be resolved, particularly in the region around the first minimum at 0.4 µs. It is likely that this is due to orientation selection effects [29] in the rigid model system. Figure 4c displays the corresponding distance distributions. The most probable distance is the same in all four distributions, and occurs at 3.4 nm. Curiously, the distribution appears to be broadened to shorter distances when using the standard configuration with *Q* ≈ 200 compared to the other distributions. This is likely to be due to slight variation in the excitation bandwidth that causes orientation selection effects to differ in this set-up.

## Discussion

A sensible PELDOR optimization requires balancing opposite factors: (i) small bandwidth (high *Q*) for efficient conversion of microwave power, (ii) large bandwidth (low *Q*) for uniform conversion at the pump and observer frequencies, (iii) large bandwidth (low *Q*) to achieve the required separation of 65 MHz between pump and observer frequency (i.e., with nitroxide spin labels).

Commercially available, single-mode resonators can span a limited range of bandwidths and, in some cases, impose a limit to the achievable separation between pump and observer frequencies. Most importantly, the resonator bandwidth significantly affects both the effective B_1_ and the relative sensitivity at the two different frequencies.

In this paper, we compare the PEDLOR performance when using two different degrees of over-coupling. According to our data, it appears that the lower *Q* ≈ 100 case is not beneficial either in terms of absolute intensity or of signal-to-noise ratio. On the contrary, the higher *Q* ≈ 200 case enhances the overall sensitivity, without compromising integrity of the measurement, in terms of loss of modulation depth. Furthermore, we showed that the pseudo-Lorentzian symmetry of the resonance could be exploited to increase the absolute signal at the observer frequency: by placing both frequencies symmetrically (*ν*_center_ ± 32.5 MHz), rather than using the standard configuration with the pump frequency located at *ν*_center_ and the observer at *ν*_center_ + 65 MHz.

To achieve optimum modulation depth, it has been noted that an inversion (pump) pulse of 12 ns is close to ideal [14]. Our strategy relies on having sufficient microwave power available to perform a proper inversion pulse away from the center of the microwave resonance. In our set-up (a standard Bruker ELEXSYS E580 spectrometer with a 1 kW TWT amplifier) using a Bruker MD5 dielectric resonator at *Q* ≈ 200, the attenuator for the pump frequency is −13 and −2 dB for the standard and symmetric configurations, respectively. As expected, more microwave power is required for the symmetric configuration to compensate for the positioning of the inversion pulse at *ν*_center_ − 32.5 MHz rather than at *ν*_center_. With *Q* ≈ 100, higher microwave power is required to maintain a 12 ns inversion (pump) pulse, and indeed for the standard configuration, it was necessary to set the attenuator for the pump frequency to 0 dB. For the symmetric configuration, even higher microwave power would be required to attain optimal inversion while maintaining a 12 ns pulse, but this could not be achieved on our spectrometer. Despite this, we note that the modulation depth for the *Q* ≈ 100 symmetric configuration is still comparable with the other data. For the Bruker split-ring MS3 resonator, which is widely used for PELDOR studies, this configuration should be easier to attain as the smaller volume means that there is more spare power available (≈6 dB) than in the MD5 resonator.

All the data presented in this paper, both EDFS spectra (Fig. 3) and PELDOR traces (Fig. 4), show a significant increase in the signal-to-noise ratio for the symmetric configuration with *Q* ≈ 200 when compared with the other configurations we have considered. Most importantly, the PELDOR shows an increase of the absolute signal by 40 %. Assuming a constant noise level, this translates into a similarly enhanced signal-to-noise ratio, which in turn corresponds to reducing the measurement time by half. We deem this to be a welcome boon for PELDOR measurements that are often painfully long.
